# Metabolomics analysis of mechanism of improving quality of *Schisandrae chinensis* fructus by NO combining with high-temperature stress

**DOI:** 10.1371/journal.pone.0327497

**Published:** 2025-07-18

**Authors:** Zhaoping Meng, Wei Zhang, Zixian Guo, Liyang Wang, Wenfei Liu, Ling Cao, Yuhua Zhang, Xiangcai Meng

**Affiliations:** 1 Department of Pharmacognosy, Heilongjiang University of Chinese Medicine, Harbin, Heilongjiang, China; 2 Heilongjiang Greater Hinggan Mountains Region Agriculture Forestry Research Institute, Jagdaqi, Heilongjiang, China; University of Brescia: Universita degli Studi di Brescia, ITALY

## Abstract

The fruits of *Schisandra chinensis* (Turcz.) Baill. (Schisandrae chinensis fructus) are a well-known herbal medicine, known for its hepatoprotective, antidepressant, antioxidant, and sedative-hypnotic properties. Over-exploitation of wild resources led to the rise of cultivation, along with a decrease in quality. Exposure of plants to adversity must generate substantial quantities of reactive oxygen species (ROS) and result in cellular damage. In response, secondary metabolites are produced to neutralize ROS; these secondary metabolites are usually the active ingredient of herbal medicine, so the quality of herbal medicine is closely related to the environment and ROS. The interplay of exogenous Nitric Oxide (NO, supplied as sodium nitroprusside) and high-temperature stress can simulate adversity and improve the quality of Schisandrae chinensis fructus; neverless, the underlying mechanism remains largely unexplored. In this study, we examined the changes in intracellular ROS levels as well as phenylalanine deaminase activities after stress and analyzed the metabolic changes using ultra performance liquid chromatography-quadrupole time-of-flight tandem mass spectrometry (UPLC-Q-TOF-MSE). The results showed that the level of superoxide anion (O_2_^_^.^_^^−^) and H_2_O_2_ increased by 25.8% and 331%, respectively, the activity of phenylalanine aminotransferase (PAL) by 69.3% on the 2^nd^ day, indicating that combination of exogenous NO with high-temperature stress could lead to physiological states of adversity stress. Twenty-two differential metabolites (VIP ≥ 1) were identified using untargeted metabolomics. 3 primary metabolites, namely mannose, pyruvate, and shikimic acid, together with 2 secondary metabolites, phenylalanine and mevalonic acid, were observed to decrease. 17 secondary metabolites, including Schisandrin A, Schisandrin B, Schisandrin C, Gomisin D, Gomisin G, Gomisin H, Benzoylgomisin H, Benzoylgomisin O,Angeloylgomisin P, Catechin, Isorhamnetin, Luteolin, Cinnamic acid, Hydroxycinnamic acid, Hexahydrocurcumin, Coniferyl alcohol, Phenylalanine, Terpinolene and Mevalonic acid, exhibited increases in their levels by 10.64, 1.84, 1.40, 1.64, 4.46, 8.18, 1.72, 10.20, 2.08, 1.27, 1.57, 1.18, 2.01, 1.12, 1.88, 1.15, and 3.17-fold, respectively. Under stress conditions, intracellular ROS levels increased, and a significant portion of primary metabolites were used for the biosynthesis of secondary metabolites with higher antioxidant activity. This redistribution of metabolic flows from basal metabolism to secondary metabolism to defend against ROS. The combination of exogenous NO with high-temperature enhances secondary metabolism of *Schisandra chinensis* fruit, which opens new avenues for production of high-quality Schisandra chinensis fructus.

## 1. Introduction

The fruits of *Schisandra chinensis* (Turcz.) Baill. (Schisandrae chinensis fructus) are a well-known herbal medicine, renowned for its hepatoprotective, antioxidant, antidepressant, sedative and hypnotic properties. Lignans such as Schizandrol A, Schizandrol B, ShiandrinA, Shiandrin B, and Shiandrin C, etc, are considered as the primary contributors to these pharmacological activities [1]. The escalating demand for Schisandrae chinensis fructus has rendered wild resources insufficient to satisfy public needs. Since the 1970s, Schisandrae chinensis fructus has been gradually converted from wild harvesting to cultivation, resulting in a deterioration of quality [2]. A comparative research of cultivated Schisandrae chinensis fructus and its wild variant revealed that the wild Schisandrae chinensis fructus has a higher lignan content and superior medicinal quality [3]. Therefore, research on the mechanisms underlying the quality formation of Schisandrae chinensis fructus quality is a primary priority.

*Yield is contingent upon a favorable environment, while quality is shaped by adversity.* The high quality of wild medicinal herbs is attributed to the adverse conditions. Adverse stress causes the closure of plant stomata, limiting the release of O_2_ in chloroplasts, the accumulated O_2_ combines with the electrons released during photosynthesis to form superoxide anion (O_2_^.^^−^), which can subsequently be converted to hydroxyl radicals (-OH), H_2_O_2_ [4]. Meanwhile, mitochondrial respiration and chloroplast photosynthesis are weakened under adverse conditions, leading to increased electron leakage, which subsequently enhances the production of O_2_^.^^−^, -OH, and H_2_O_2_ [5]. These highly reactive oxygen-containing compounds are also known as reactive oxygen species (ROS). ROS are essential for plant life, act as signaling molecules in cell proliferation, differentiation, and apoptosis. However, high level of ROS has strong oxidizing ability, which can destroy protein structure, damage DNA, interfere with metabolism, and even lead to death [4]. Over the course of long-term evolution, plants have developed a biological mechanism to eliminate excessive ROS. Once ROS are over-produced, their content or activity increases, triggering a negative feedback process and maintains a relatively stable level. The antioxidant defense system of plants mainly contains two parts: antioxidant enzymes such as superoxide dismutase (SOD), catalase (CAT), peroxidase (POD), etc., and secondary metabolites such as flavonoids, lignans, terpenoids, etc [6]. However, antioxidant enzymes are also proteins that are structurally unstable and highly susceptible ROS induced damage. Therefore, they alone are not sufficient to adapt plants to severe stress conditions. Secondary metabolites play a crucial role as antioxidants, helping to neutralize ROS or mitigate ROS-induced cellular damage. Due to their antioxidant, anti-inflammatory and antibacterial properties. They are also the main active ingredient in herbal medicines. Therefore, the ability of adversity to improve the quality of herbs stems is closely linked to ROS [7]. Studies have shown that constructing the physiological state of plants under adversity through ROS can promote the secondary metabolism, and improve the quality of herbal medicine [2]. H_2_O_2_ increased cannabidiol (CBD) content of *Cannabis sativa* flower cluster by 72.9% [8], exogenous sodium nitroprusside (SNP) increased the four active components of *Saposhnikovia divaricata* (Turcz.)Schischk,prim-O-glucosylcimifugin, cimifugin, 4-O-β-d-glucosyl-5-O-methylvisamminol, and sec-O-glucosyl-hamaudol, increased by 88.3%, 325.0%, 55.4%, and 283.8%, respectively [9]; produced ROS by high-temperature increased the content of Liquiritigenin and Isoliquiritigenin in licorice by up to 86.46% and 150.00% [10]. Study also has showed that high-temperature also promoted the production of ROS and increased the content of five lignans of Schisandrae chinensis fructus by 31.2%~81.5%, respectively [11].

Efforts to improve herb quality through ROS have been fruitful, but little is known about the overall effects of exogenous ROS on plant metabolism. Secondary metabolites are the main active ingredients of medicinal plants, exhibiting diverse components, significant variations in activity, and unstable content. Metabolomics can reveal interactions between all small molecule metabolites in organisms at different physiological states, environmental conditions, or species through quantitative and qualitative analyses of metabolites [12], thereby clarifying the mechanism of herb quality formation. Nitric oxide (NO) promotes stomatal movement, prevents apoptosis, and regulates ROS production, gaining significant attention as a key signaling molecule for plant adaptation to biotic and abiotic stresses [13]. It can modulate the activity of Ca^2+^ channels and MAPK to produce ROS, also react with O_2__^^.^ ^_^−^ to produce peroxynitrite (ONOO-) of low oxidizability [14,15], or act as an antioxidant to eliminate ROS [16], hence exhibiting dual efficacy without causing additional cellular damage. High-temperature, a common environmental stress in plants, can cause an increase in intracellular O_2_^.^^−^, H_2_O_2_, and malondialdehyde (MDA), resulting in an increase in membrane permeability, electrolyte leakage, and ultimately cell damage [17]. NO enhances plant antioxidant capacity by activating antioxidant enzymes (APX, CAT, SOD) during high-temperature stress, demonstrating its role in high-temperature stress response [18].

In summary, wild Schisandrae chinensis fructus often encounters various stresses, resulting in better medicinal quality, primarily through the generation of ROS. Therefore, in this study, the ROS level was artificially elevated by combining exogenous NO and high-temperature to simulate the physiological state of Schisandrae chinensis fructus under unfavourable conditions.

## 2. Materials and instruments

### 2.1. Materials

Fresh fruits of *Schisandrae chinensis* were purchased from Daxinganling Arctic Schisandra Science and Technology Co. Ltd (Baiyinna Township, Huma County) in September 2023, and were identified by Prof. Meng Xiangcai of Heilongjiang University of Traditional Chinese Medicine.

### 2.2. Instruments

Waters Acquity TM UPLC liquid chromatograph (Waters Corporation, USA); Synapt TM G2-Si mass spectrometry system (Waters Corporation, USA), MassLynx V4.2 Workstation (Waters Corporation, USA), ACQUITY UPLC BEH C18 column (Waters Corporation, USA), Cryogenic ultra-high speed centrifuge ThermoSorvall ST16R (Thermo, USA), Progenesis QI V3.0 (Waters, USA), Heto Power Dry PL3000 lyophilizer (Thermo, USA);Electronic analytical balance (Shanghai Puchun Metrology Instrument Co., Ltd.)

### 2.3. Reagents

Quantitative protein (TP) kit (Nanjing Jianjian Biological Company, 20230420), H_2_O_2_ kit (Nanjing Jianjian Biological Company, 20230726), phenylalanine deaminase (PAL) kit (Nanjing Jianjian Biological Company, 20230403), Intra-assay Coefficient of Variation (CV): 1.2%;Inter-assay Coefficient of Variation (CV): 2.35%; Limit of Detection (LOD): 0.5 mol/L,Detection Range: 0.5–386.3 mol/L. O_2__^^.^^_^−^ radical assay kit (Beijing Solepol Technology Company, 2309002) LOD: 0.001451 µmol/ml. Leucine-enkephalin (SIGMA, USA), Chromatography grade methanol, acetonitrile (Merck, Germany), Formic acid, chromatography grade (Komio Chemical Reagent, China), Analytical grade methanol (Beijing Fine Chemicals Co., Ltd., Beijing)

## 3. Methods

### 3.1. Sample handling

On September 5, 2023, the harvested Schisandrae chinensis fructus samples were randomly assigned to 2 groups. Based on the results of the previous experiments, the treatment group of *Schisandrae chinensis* fresh fruits were immersed in an aqueous solution of 0.5 mmol/L SNP for 24 h, and then placed in a thermostat at 35°C. For the blank group, samples were maintained at room temperature without any treatment. All samples in both groups were covered with gauze and sprayed with water 3–5 times daily to prevent dehydration. Samples were collected at 0, 1, 2, and 3 d for further analysis. The following methods were used: (1) The pericarp and pulp from the same cluster *Schisandrae chinensis* were removed, and 0.3 g was precisely weighed. The samples were pre-frozen at −80°C for 46 h and then freeze-dried at −40°C for 48 hunder a vacuum of 20 Pa. The sample was lyophilized and kept independently for metabolomic analysis. Untreated samples stored at room temperature on day 0 were used as controls for metabolomics assays. The samples were replicated 5 times. (2) Fresh fruits of *Schisandra chinensis,* from which the pericarp and pulp were removed, were used for seed collection*,* and several 0.3 g portions of seeds were weighed precisely, immediately frozen in liquid nitrogen, and stored at −80°C for later determination of O₂^.^^⁻^ and H₂O₂ content, as well as PAL activity. The blank group served as a control.

### 3.2. ROS level measurement

The protein concentration in the seeds of *Schisandrae chinensis* was detected according to the instructions of the Quantification of Protein (TP) assay kit, and the level of TP was expressed as g/L (unit). The intracellular levels of O_2_^.^^−^ and H_2_O_2_ were determined according to the O_2_^.^^−^ and H_2_O_2_ kit instructions. The levels of O_2_^.^^−^and H_2_O_2_ were expressed as μmol·g^−1^ (unit) and mmol·gpot^−1^ (unit), respectively. Samples from 3 independent plants were tested in three technical replicates, and the technical replicate data were averaged to represent the final value for that biological replicate. Statistical analysis was carried out based on biological replicates (n = 3).

### 3.3. Measurement of phenylalanine deaminase (PAL) activities

PAL activity was determined according to the kit instructions and expressed as U/g (unit).

### 3.4. Preparation of test material

Lyophilized *Schisandra chinensis* seeds were ground into a fine powder, and 250 mg of the powder was placed into a conical flask. Subsequently, 10 ml of 70% methanol was added, and the flask was weighed and sealed with a sealing film. The mixture was heated at 60°C in a water bath for 20 min, and any weight loss was compensated. The resulting aqueous solutions were centrifuged at 10,000 rpm for 10 min, and the supernatant was filtered through a microporous filter membrane (0.22 μm pore size) into the injection vial for subsequent LC-MS/MS analysis. Another equal amount of each test solution was aspirated 400 μL, mixed well, and prepared as a quality control (QC) sample to monitor the stability of the analytical system and method.

### 3.5. UPLC/QTOF-MS

Waters ACQUITY UPLC BEH C_18_ column (100 mm x 2.1 mm, 1.7 µm) pre-columned with AC-quality UPLC® BEH Shield RP-18 1.7 µm. The mobile phases were 0.1% formic acid acetonitrile solution (A) and 0.1% formic acid aqueous solution (B), and the gradient elution was as follows: 0 ~ 1 min, 1% ~ 99% (A); 1 ~ 9 min, 60% ~ 40% (A); 9 ~ 14 min, 65% ~ 35% A; 14 ~ 17 min, 70% ~ 30% A; 17 ~ 20 min, 95% ~ 5%; 20 ~ 21 min, 99% to 1%, 21–22 min, 1% to 99% (A). Column temperature 40°C, autosampler temperature 8°C, injection volume 2 µL, flow rate 0.3mL/min. Column temperature 40°C, autosampler temperature 8°C, injection volume 2 µL, flow rate 0.3mL/min.

Data were collected using a Waters Synapt G2-Si MS mass spectrometry system in both positive (ESI+) and negative (ESI-) ion modes,with an electrospray ionization source (ESI). The ionization source temperature was set to 110°C, the desolventization gas temperature to 400°C, the desolventization gas flow rate of 800 L/h, and the cone pore gas flow rate to 50 L/h. The extraction cone pore voltage was 4.0 V, and the capillary was 3 kV in positive ion mode and 2.5 kV in negative ion mode. The sample cone voltages were 30 V and 40 V for positive and negative ion modes, respectively. Leucine-enkephalin([M+H]^+^556.2771, [M+H]^−^554.2615) was used as the lock mass compound. The data were collected in MSE mode with an acquisition time of 22.0 min, a mass scan range of m/z 50–1200, and a scan time of 1.0 s. Mass spectrometry data were collected with a low collision energy of 4 V, a high collision energy of 15~40 V for positive ions, and a high collision energy of 10–30 V for negative ions.

### 3.6. Data acquisition and analysis

Data acquisition was performed through a Mass Lynx V4.2 workstation, and Progenesis QI analysis software was applied to analyze the mass spectral data. In the positive ion mode, the compounds were detected as [M+H]^+^, [2M+H]^+^, [M+Na]^+^. In the negative ion mode, the compounds were detected as [M-H]^−^, [M+FA-H]^−^, [2M-H]^−^. The component database of Schisandrae chinensis fructus, created using PubChem website and Progenesis SDF Studio software, was compared with the mass spectrometry data for rapid screening. Subsequently, the retention time, molecular weight, and secondary fragments of the components were verified based on literature reports or the PubChem database to finalize the identification of the components. Charts were plotted using Origin 2021, SIMCA (14.1), and AI.

### 3.7. Statistical analysis

Statistical analysis was conducted using IBM SPSS Statistics (version 27.0, IBM Corporation, Armonk, NY, USA). Independent samples t-tests were performed to compare the levels of O_2_^.^^−^, H_2_O_2_, PAL, and differential metabolites between days 1, 2, and 3 and day 0. A two-tailed test was used, and statistical significance was set at p < 0.05. The false discovery rate (FDR) in the metabolomic data was adjusted for multiple comparisons using the Benjamini-Hochberg procedure. Differential metabolites were identified based on an FDR-adjusted p-value (q-value) threshold of < 0.05.

## 4. Results and analysis

### 4.1. Effect of stress on ROS metabolism

**Fig 1:**
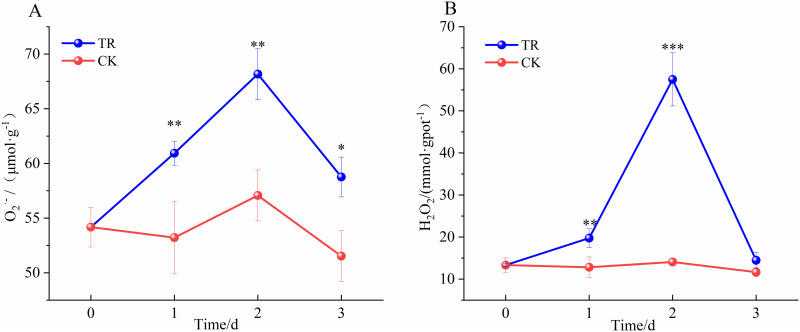
O_2_^.^^−^ (A) and H_2_O_2_ (B) levels under NO and high-temperature stress.

The effects of exogenous NO combined with high-temperature on the ROS levels of *Schisandrae chinensis* are shown in Fig 1. O_2_^.^^−^ and H_2_O_2_ showed a trend of increasing and then decreasing, and both peaking on day 2. O_2_^.^^−^ increased by 25.8% on day 2 (independent samples t-test, t(4) = −8.202, p = 0.001), and H_2_O_2_ increased by 331.2% on day 2 (independent samples t-test, t(4) = −4.907, p = 0.008) compared to day 0.

### 4.2. Effect of stress on phenylalanine deaminase (PAL) activity

**Fig 2:**
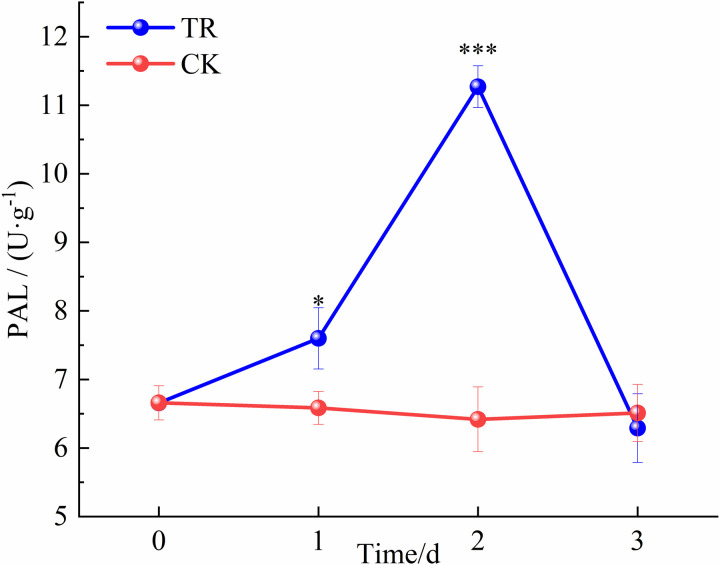
PAL activities under NO and high-temperature stress.

PAL activities showed an initial increase followed by a decrease, with a rise of 14.1% (independent samples t-test*, t*(4) = −3.178, *p* = 0.034) on the first day and 69.3% (independent samples t-test, *t*(4) = −20.281, *p* = 0.000035) on day 2, as shown in Fig 2.

### 4.3. Sample quality control analysis

**Fig 3:**
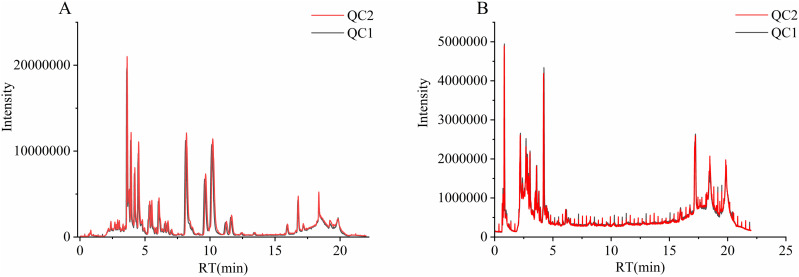
Overlay plot of TIC for mass spectrometry detection of QC samples. A: positive ion mode, B: negative ion mode.

Quality control (QC) samples have been mixed with all other samples. During the analysis, one quality control sample was introduced for every five samples examined to assess the instrument’s stability. As shown in Fig 3, the total ion chromatogram (TIC) for all QC samples was overlaid, indicating high instrumental stability and data reliability.

### 4.4. Identification of chemical composition

**Fig 4:**
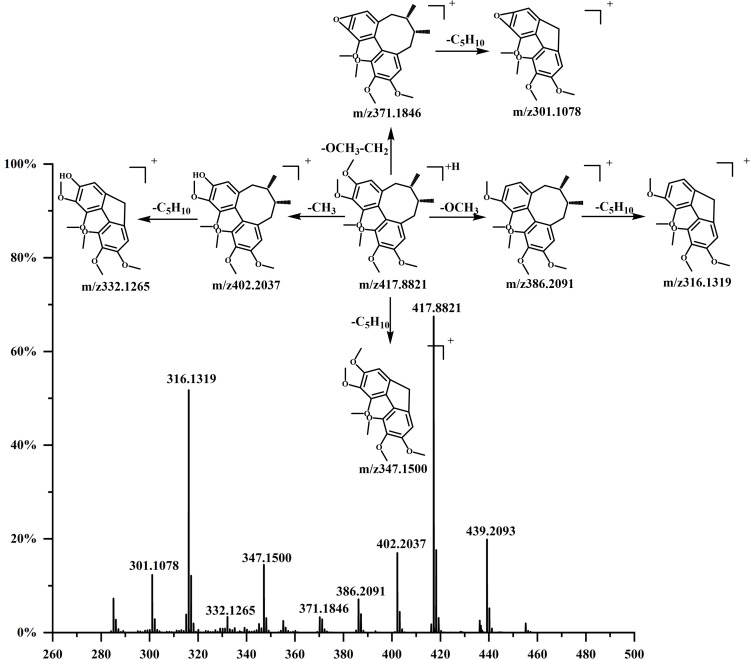
Fragmentation patterns for Schisandrin A.

The chromatograms of the test material were tested both in positive and negative ion modes, according to the method under 3.5. The data were analyzed using the method described in Section 3.6, with Schisandrin A as a representative example. Fig 4 showed that analysis by Progenesis QI software matches the ion peak at m/z 439.2093 [M+Na]^+^ at 7.77 min. The peak at m/z 417.8821 undergoes a -CH_3_ loss to form a fragment ion at m/z 402.2037, a -OCH_3_ loss to form a fragment ion at m/z 386.2091, a -OCH_3_-CH_2_ loss produces fragment ion of m/z 371.1846, and the loss of -C_5_H_10_ gives a fragment ion of m/z 347.1500. The fragment ion at m/z 402.2037 undergoes a -C_5_H_10_ loss, producing m/z 332.1265. Likewise, m/z 386.2091 eliminates -C_5_H_10_, forming m/z 316.1319, while m/z 371.1846 experiences the same loss, generating m/z 301.1078. The cleavage process was highly consistent with the literature [19], hence it was identified as Schisandrin A. A total of 30 compounds in *Schisandrae chinensis* seeds were identified based on the above method, the details are shown in Table 1.

**Table 1:** Characterization of chemical constituents in seeds of *Schisandrae chinensis.*

No	Rt (min)	Selected ion	Measured mass (m/z)	Calc. mass (m/z)	Error (ppm)	MS/MS fragment ion (m/z)	Formula	Identification
1	0.81	[M+H]^+^	181.055	181.0501	27.1	164.0411, 145.0620	C_9_H_8_O_4_	Caffeic acid
2	0.83	2[M-H]^−^	119.0352	119.0344	6.7	101.0246, 93.0353	C_2_H_4_O_2_	Acetic Acid
3	0.83	[M-H]^–^	191.056	191.0556	2.1	146.0456, 96.9632	C_7_H_12_O_6_	Quinic acid
4	0.84	[M-H]^–^	149.0452	149.045	1.3	146.0461, 131.0353	C_5_H_10_O_5_	Arabinose
5	0.85	[M-H]^–^	173.0451	173.045	0.6	157.4465, 141.9333, 129.0189	C_7_H_10_O_5_	Shikimic acid
6	1.19	[M-H]^–^	87.001	87.0082	20.7	86.1270, 59.0111	C_3_H_4_O_3_	Pyruvic Acid
7	2.08	[M+H]^+^	317.0659	317.0661	−0.6	245.0401, 217.0619, 203.0370, 153.0558	C_16_H_12_O_7_	Isorhamnetin
8	2.19	[M+H]^+^	291.0887	291.0869	6.2	165.0885, 139.0408, 123.0458	C_15_H_14_O_6_	Catechin
9	2.20	[M-H]^–^	151.0405	151.0395	6.6	123.0454, 108.0219	C_8_H_8_O_3_	Vanillin
10	2.31	[M-H]^–^	463.0921	463.0877	9.5	447.0657, 429.0421, 369.1166, 345.1064	C_21_H_20_O_12_	Hyperoside
11	2.86	2[M+H]^+^	217.122	217.1229	−4.1	107.0880, 77.0406	C_7_H_8_O	4methylphenol
12	3.00	2[M+H]^+^	359.1494	359.1495	−0.3	161.0972, 146.0966	C_10_H_12_O_3_	Coniferyl alcohol
13	3.28	[M+Na]^+^	553.2052	553.205	0.4	401.1844, 383.1497, 353.1359, 341.1046, 337.1101	C_28_H_34_O_10_	Gomisin D
14	3.34	[M+Na]^+^	441.1883	441.1889	−1.4	370.1778, 355.1549, 323.1291, 201.0915	C_23_H_30_O_7_	Gomisin H
15	3.63	[M-H]^–^	121.0298	121.0290	6.6	108.0166, 93.3130	C_7_H_6_O_2_	4-hydroxybenzaldehyde
16	4.40	[M+Na]^+^	545.2140	545.2151	−2	508.2074, 493.1767, 409.1732	C_30_H_34_O_8_	Benzoylgomisin H
17	4.74	[M+H]^+^	385.1647	385.1651	−1	355.1541, 285.0775, 257.0775, 227.0707	C_22_H_24_O_6_	Schisandrin C
18	5.33	[M+Na]^+^	559.2045	559.2062	−3	415.1756, 371.1512, 342.1099	C_30_H_32_O_9_	Gomisin D
19	5.49	2[M+H]^+^	297.1142	297.1127	5	250.0593, 249.1075, 242.0807	C_9_H_8_O_2_	Cinnamic acid
20	6.51	2[M+H]^+^	329.1017	329.1025	−2.4	311.0944, 283.0981, 255.0644	C_9_H_8_O_3_	Hydroxycinnamic acid
21	6.52	[M+H]^+^, [M+Na]^+^	515.2283	515.2281	0.4	537.2093	C_28_H_34_O_9_	Angeloylgomisin P
22	8.15	[M+Na]^+^	439.2093	439.2097	−0.9	417.2281, 405.2035, 402.2037, 386.2091, 371.1846, 347.1500, 332.1265, 316.1319, 301.1078	C_24_H_32_O_6_	Schisandrin A
23	8.63	[M-H]^–^	149.0118	149.0086	21.5	103.3838, 87.0793	C_4_H_6_O_6_	L-Tartaric acid
24	9.02	[M+H]^+^,[M+Na]^+^	401.1957	401.1964	−1.7	386.1726, 370.1781, 300.1000, 258.0775, 227.0706	C_23_H_28_O_6_	Schisandrin B
25	9.02	[M+Na]^+^	397.1649	397.1627	5.5	379.1677, 199.0700, 159.0769	C_21_H_26_O_6_	Hexahydrocurcumin
26	9.63	[M+H]^+^	245.0737	245.0774	−15.1	209.0460, 191.0737, 179.1719	C_9_H_12_N_2_O_6_	Pseudouridine
27	12.40	[M+Na]^+^	543.1995	543.1997	0.4	421.1629, 399.1814, 368.1625, 357.1333	C_30_H_32_O_8_	Benzoylgomisin O
28	18.70	[M-H]^–^	179.0543	179.0556	−19.3	89.0235	C_6_H_12_O_6_	Mannose
29	19.15	[M+FA-H]^–^	210.0787	210.0766	10	193.0827, 166.0745, 147.1204	C_9_H_11_NO_2_	Phenylalanine
30	19.67	[M-H]^–^	147.0674	147.0657	11.6	111.0471, 69.0109, 57.5710	C_6_H_12_O_4_	Mevalonic acid

### 4.5. Multivariate statistical analysis

#### 4.5.1. Principal components analysis.

**Fig 5:**
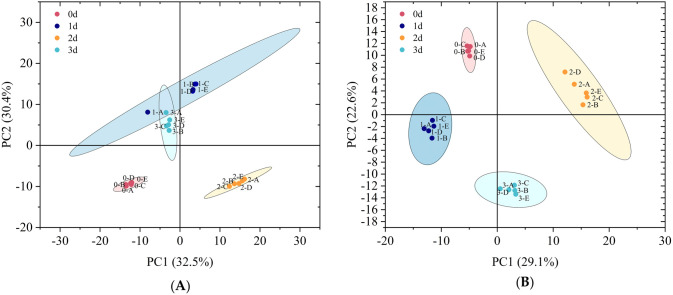
Inter-group differences in positive (A) and negative (B) ion modes. 0, 1, 2, and 3 are the number of days, and 0 is the control group.

Principal Component Analysis (PCA) is a commonly used multivariate data analysis method for unsupervised pattern recognition, which extracts feature components from high-dimensional data, transforms them into low-dimensional data, and visualizes them in 2D or 3D plots. Fig 5 showed that the five replicates of the same group were well clustered, indicating low intergroup variability and high data quality reliability. Principal component 1 (PC1) and principal component 2 (PC2) account for 32.5% and 30.4%, respectively, in the positive ion mode, and 29.1% and 22.6%, respectively, in the negative ion mode. There was a clear trend of segregation between groups, indicating significant differences in metabolites after stress.

#### 4.5.2. Orthogonal partial least squares-discriminant analysis.

Orthogonal partial least squares-discriminant analysis (OPLS-DA) is a multivariate statistical analysis method for supervised pattern recognition. It removes irrelevant variation to identify differential metabolites, and has also been widely used to distinguish between different populations.

**Fig 6:**
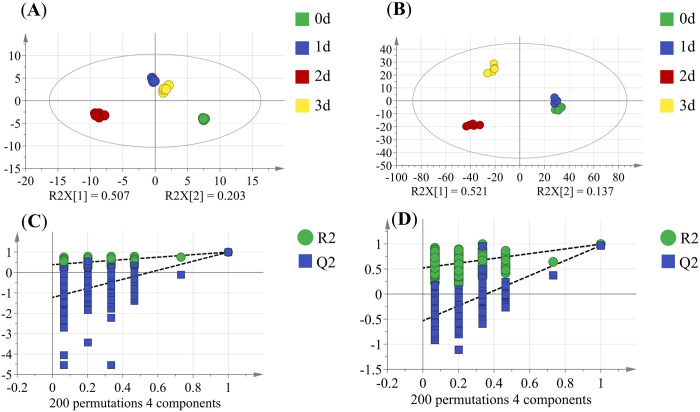
Plots of OPLS-DA and permutation test in positive (A, C) and negative (B, D) ion modes. 0, 1, 2, and 3 are the number of days, and 0 is the control group.

As shown in Fig 6, the OPLS-DA model exhibited a fit index (R_x_^2^) of 0.98 for the independent variable, a fit index (R_y_^2^) of 0.995 for the dependent variable, and the model prediction index (Q^2^) of 0.982 in the positive ion model. In the negative ion model, R_x_^2^, R_y_^2^, and Q^2^ were 0.78, 0.995, and 0.967, respectively, all exceeding 0.5, indicating that the model was well constructed and had a reliable prediction. A 200 permutation test (i.e., 200 permutation experiments) on R^2^ and Q^2^ showed that the intercept of Q^2^ on the Y-axis is less than 0, indicating that the model is not overfitted.

The previous experiments found that the contents of secondary metabolites peaked on the 2nd day of stress. Consequently, we used OPLS-DA analysis to compare the 2d stress group with the control group, thereby obtaining the VIP value of each metabolite in the sample. Then we screened the differential metabolites according to the univariate statistics P-value or Fold change. In this study, we screened fold change ≥ 2 or fold change ≤ 0.5 and VIP ≥ 1 as the most significant differential chemical markers.

**Fig 7:**
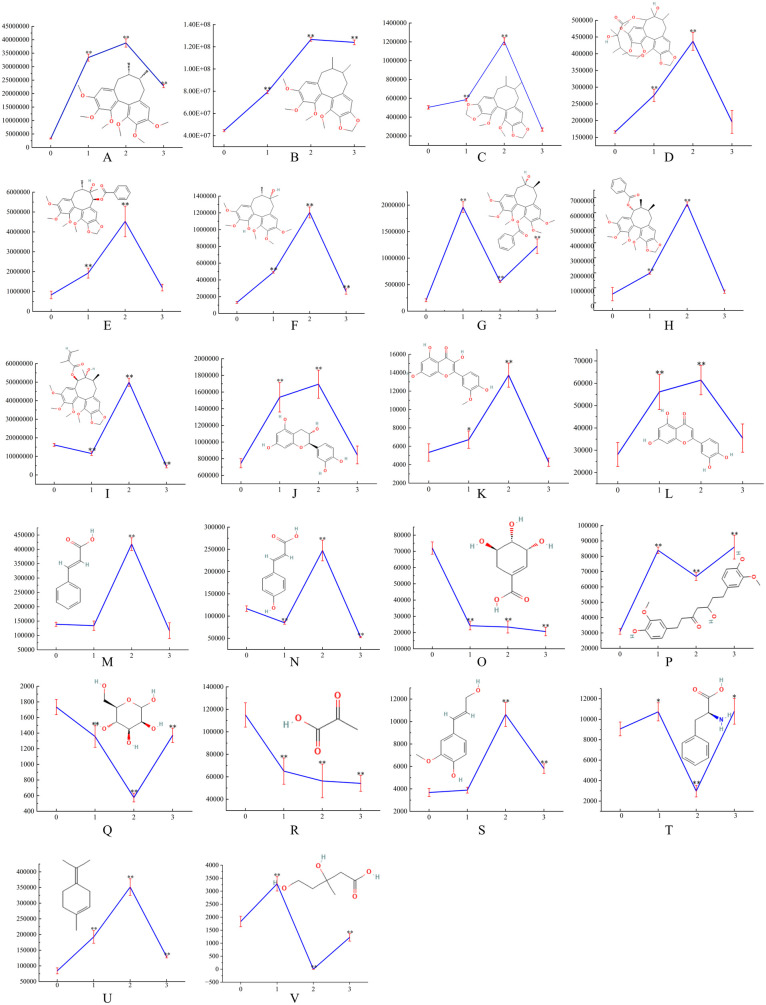
22 differential metabolites between the stress group and the control. A: Schisandrin A; B: Schisandrin B; C: Schisandrin C; D: Gomisin D; E: Gomisin G; F: Gomisin H; G: Benzoylgomisin H; H: Benzoylgomisin O; I: Angeloylgomisin P; J: Catechin; K: Isorhamnetin; L: Luteolin; M: Cinnamic acid; N: Hydroxycinnamic acid; O: Shikimic acid; P: Hexahydrocurcumin; Q: Mannose; R: Pyruvic acid; S: Coniferyl alcohol; T:Phenylalanine; U: Terpinolene; V: Mevalonic acid. 0, 1, 2, and 3 represent the number days.

A total of 22 differential metabolites were identified by comparing the 2d stress group with the control group, comprising 19 secondary metabolites, including Schisandrin A, Schisandrin B, Schisandrin C, Gomisin D, Gomisin G, Gomisin H, Benzoylgomisin H, Benzoylgomisin O, Angeloylgomisin P, Catechin, Isorhamnetin, Luteolin, Cinnamic acid, Hydroxycinnamic acid, Coniferyl alcohol, Hexahydrocurcumin, Phenylalanine, Terpinolene and Mevalonic acid, and the remaining 3 were primary metabolites, including Mannose, Pyruvic acid, and Shikimic acid (As shown in Fig 7). The peak areas of each differential metabolite on days 1, 2, and 3 were compared to day 0 using an independent sample t-test, with statistical significance set at *p* < 0.05.

#### 4.5.3. Correlation analysis.

**Fig 8:**
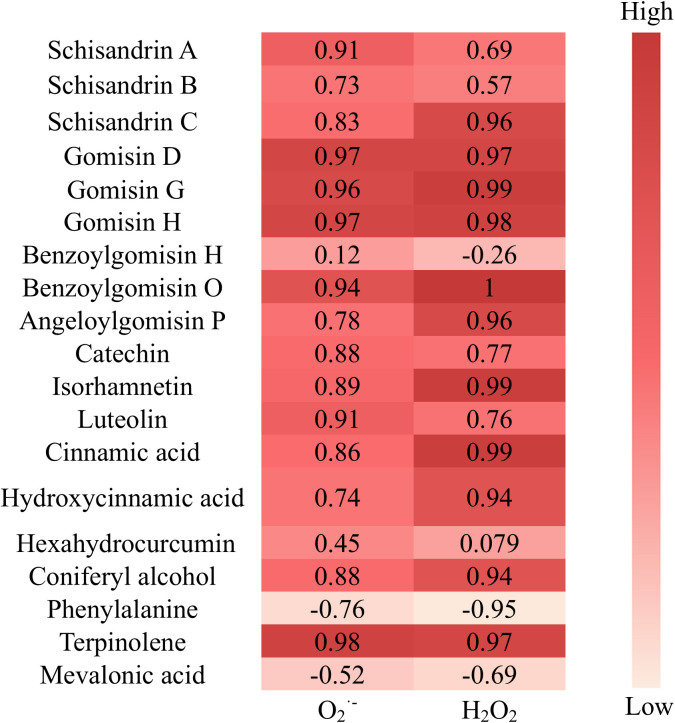
Correlation heatmap of O_2_^.^^−^, H_2_O_2_, and differential secondary metabolites.

The correlation between O_2_^.^^−^, H_2_O_2_, and differential secondary metabolites was examined using Pearson correlation heatmap analysis, revealing robust correlations between ROS and secondary metabolites, with correlation coefficients > 0.9 (*p* < 0.01) for O_2_^.^⁻ with Terpinolene, Gomisin D, Gomisin H, Gomisin G, Benzoylgomisin O, Schisandrin A, Luteolin, and coefficients > 0.9 (*p* < 0.01) for H_2_O_2_ with Benzoylgomisin O, Gomisin G, Isorhamnetin, Cinnamic acid, Gomisin H, Gomisin D, Terpinolene, Schisandrin C, Angeloylgomisin P, Hydroxycinnamic acid, Coniferyl alcohol. As shown on Fig 8.

#### 4.5.4. PLSR analysis.

Partial least squares regression (PLSR) analysis was performed to explore the correlation between ROS and secondary metabolites, using O_2_^.^^−^ and H_2_O_2_ as independent variables and 19 secondary metabolites as dependent variables. The high R²X value (96.1%) indicated that the model effectively captures ROS variability, while the R²Y value (72.3%) suggested a significant association between ROS and the variability in secondary metabolites. The Q² value (0.503), exceeding the threshold of 0.5, confirmed the model’s strong predictive ability.

**Fig 9:**
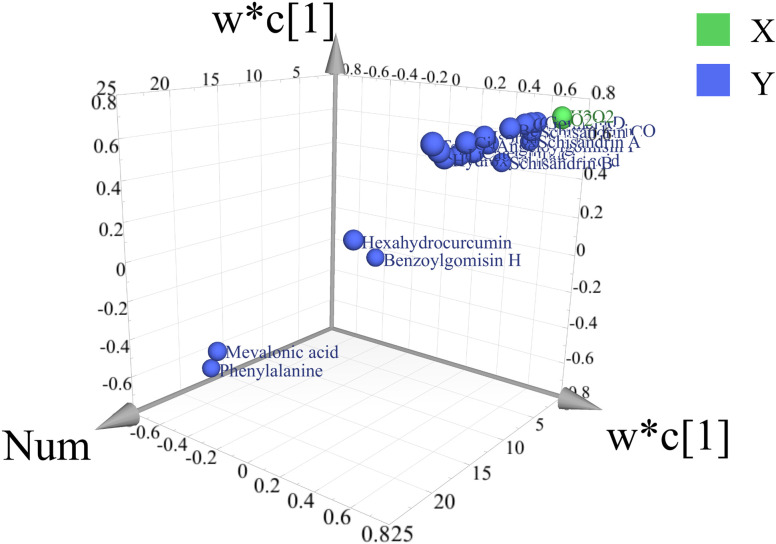
3D loading plot of secondary metabolites relative to ROS in the PLSR.

**Fig 10:**
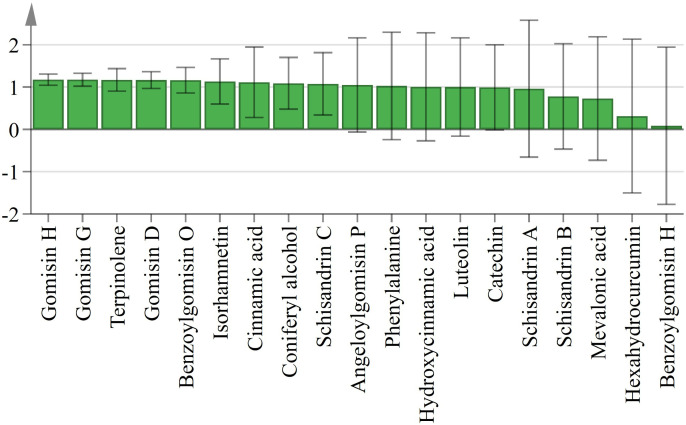
VIP scores of second metabolites in the PLSR model.

Fig 9 shows the spatial distribution of secondary metabolites related to the two principal components (X and Y), with green markers representing O_2__^^.^^_^−^ and H_2_O_2_. Metabolites located near the ROS markers, such as Gomisin G, Gomisin H, show strong correlations, whereas those farther away are less affected. This pattern illustrates the association between ROS and secondary metabolite accumulation. The VIP scores quantify the contribution of each secondary metabolite to the PLSR model. As shown in Fig 10, the 5 metabolites with the highest VIP scores are Gomisin G, Gomisin H, Terpenoids, Gomisin D, and Benzoylgomisin O.

## 5. Discussion

The quality of herbal medicines has been *adversely affected*, and it has been proved that moderate adversity promotes an increase of secondary metabolite contents in many medicinal plants. For instance, high-temperature increased the levels of Liquiritigenin, Isoliquiritigenin, Liquiritin apioside, and Isoliquiritin apioside of *Glycyrrhiza uralensis* by 86.46%, 150.00%, 76.06%, and 100.00%, respectively, while also promoted the biosynthesis of artemisinin by promoting the expression of synthetase genes. Drought increased the contents of Atractylodin, β-Eudesmol, Atractylon and 2-Atractylenolide in *Atractylodis sinensis* by 51.0%, 36.9%, 47.1%, and 91.5%, respectively. It has been demonstrated that *Schisandrae chinensis* also exhibits an “adversity effect”, whereby exposure to high-temperature at 35°C effectively the biosynthesis of its secondary metabolites: Schisandrol A, Schisandrol B, Schisandrin A, Schisandrin B, and Schisandrin C, with increases of 48.0%, 64.6%, 81.5%, 56.8%, and 31.2%, respectively. This experiment utilized exogenous NO combined with elevated temperatures to investigate more effective strategies for improving the quality of Schisandrae chinensis fructus. Pre-experimental results showed that the single high-temperature group showed increases of 10.3%, 49.0%, 14.4%, 42.5%, and a decrease of 14.3% for Schisandrol A, Schisandrol B, Schisandrin A, Schisandrin B, and Schisandrin C, respectively;0.1 mmol/L SNP increased these metabolites by 29.0%, 1.9%, 134.3%, 16.9%, and 15.0%; 0.5 mmol/L SNP by 50.3%, 83.7%, 47.9%, 99.6%, and 139.3%; and 2.0 mmol/L SNP by 29.4%, 75.3%, 13.1%, 72.5%, and 44.2%, respectively, which demonstrated that the combination of SNP and high-temperature exhibited superior effects, and compared to single high-temperature group, the effect observed in the NO combined with high-temperature group is more significant, further confirming the unique role of NO. In summary, this study selected the combination of 0.5 mmol/L SNP with 35°C high-temperature.

The secondary metabolites of Schisandrae chinensis fructus are mainly found in the seeds, while the pericarp and pulp contain minimal secondary metabolites, primarily composed of polysaccharides and other nutrients [20]. Therefore, the seeds of Schisandrae chinensis fructus were used for metabolomics analysis. The results showed that a total of 22 differential secondary metabolites were observed in the stress group for the 2^nd^ day compared to the control group. Among these, 18 were secondary metabolites, including lignans, flavonoids, simple phenylpropanoids, and terpenoids, while the remaining 3 were primary metabolites, including Mannose, Pyruvic acid, and Shikimic acid.

### 5.1. Effects of stress on O_2_^_^.^_^^−^ and H_2_O_2_

Plants produce large amounts of ROS under adverse conditions, and excessive ROS can lead to oxidative damage. Exogenous NO and high-temperature stress increased the levels of O_2_^.^^−^ and H_2_O_2_, indicating that NO along with high-temperature, can mediate the production of ROS. The ROS level decreased in the later stage, likely due to the activation of the plant’s antioxidant system, which eliminated the excess ROS. The combination of NO and high-temperature triggered the over-production of ROS, inducing a physiological state in Schisandrae chinensis fructus under diversity, leading to multiple changes in physiology and metabolism, thus creating a strong correlation between ROS and secondary metabolites.

### 5.2. Effects of stress on secondary metabolism

Many secondary metabolites possess aromatic, highly conjugated -OH, -NH_2_ and methoxy (-OCH_3_) groups, that can eliminate ROS. Phenolic compounds are defined by the substitution of a hydrogen atom on the aromatic ring with a hydroxyl group or a functional derivative [21], including lignans, flavonoids, phenolic acids, tannic acid, etc., which exhibit antioxidant effects. Their antioxidant properties are mainly achieved by scavenging ROS, inhibiting of enzymes, or chelating of certain metal ions to inhibit ROS generation or to enhance the antioxidant system [22].

**Fig 11:**
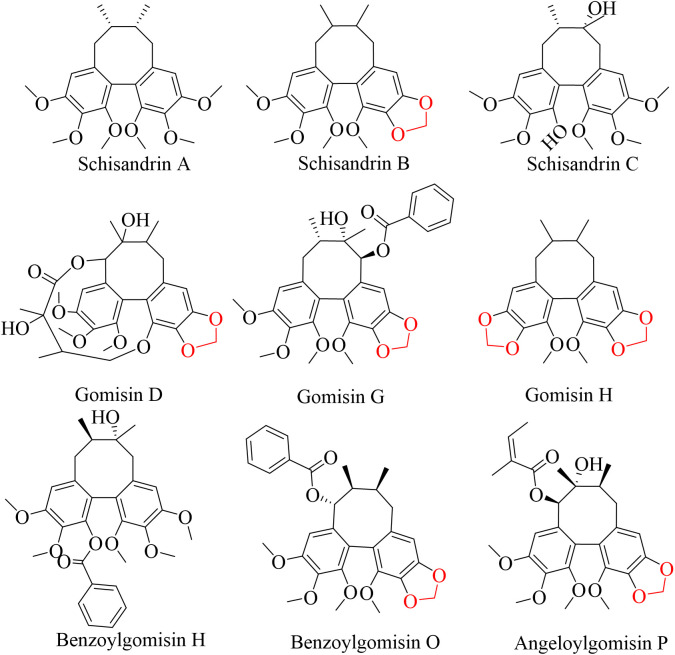
Chemical structure of a lignan.

Lignans are the main active components of Schisandrae chinensis fructus. Schisandrin A, Schisandrin B, Schisandrin,Gomisin D, Gomisin G, Gomisin H, Benzoylgomisin H, Benzoylgomisin O, and Angeloylgomisin P are classified as biphenyl cyclooctenyl lignans, as shown in Fig 11. They comprise of two benzene rings interconnected by a carbon bridge, featuring substituents such as methoxy(-OCH_3_), hydroxyl(-OH), methyldioxy(-O-CH_2_-O-), acetyl(CH_3_CO-), benzoyl, and angelicinoyl. These compounds can exhibit antioxidant effects by activating antioxidant pathways, improving antioxidant enzyme activities, and reducing ROS levels, all of which are inextricably linked to their aromatic structures and substituents [23]. This research identifies 19 different secondary metabolites, comprising 9 lignan components, all of which usually contain several antioxidant-related -OCH_3_ groups [24]. In fact, the ability of -OCH_3_ to eliminate ROS is inherently weak; its reaction with ROS requires catalysis by POD [25], indicating that the activity of -OCH_3_ is partially dependent on POD. POD is an enzyme that is induced by adversity, with its gene expression and activity regulated by ROS, usually having high activities in their presence [26]. Consequently, elevated lignan components have high antioxidant effects under adverse conditions. In addition, methylene dioxygen in lignans plays an important role in promoting the antioxidant effects of mitochondrial glutathione (GSH), which significantly enhances lignans’ bioactivity [23]. Therefore, increased lignan levels can attenuate ROS damage and improve cellular adaptation to adversity.

**Fig 12:**
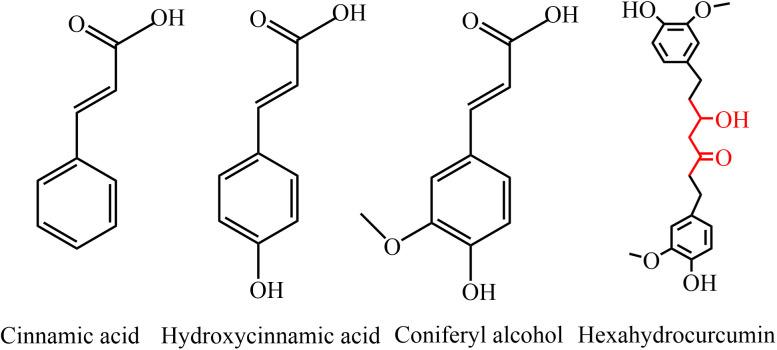
Cinnamic acid, hydroxycinnamic acid, coniferyl alcohol, hexahydrocurcumin structure.

Cinnamic acid, p-hydroxycinnamic acid, and coniferyl alcohol are phenolic acids, characterized by the presence of -OH, -COOH, and benzene rings, featuring a highly conjugated structure with potent antioxidant capacity [27]. In particular, Coniferyl alcohol is formed by the reduction of the carboxyl group (-COOH) in cinnamic acid, together with the addition of a -OH and -OCH_3_ group to the benzene ring, resulting in higher antioxidant properties. Hexahydrocurcumin is also one of the major metabolites of the polyphenolic compound curcumin. Its -OCH_3_ group enhances its ability to eliminate ROS [24], and the 3-hydroxy-1-one moiety may also exhibit greater antioxidant capacity compared to the conjugated alkenones of other curcumin analogs, thus exhibiting more potent antioxidant activity than curcumin itself [28,29]. In addition, Hexahydrocurcumin also increases the activity of antioxidants such as GSH and SOD, while promoting the elimination of ROS [30]. The chemical structures of Cinnamic acid, Hydroxycinnamic acid, Coniferyl alcohol, and Hexahydrocurcumin are shown in Fig 12 Furthermore, as shown in Fig 14, Cinnamic acid, Hydroxycinnamic acid, and Coniferyl alcohol, which serve as key intermediates in the biosynthesis of lignans and flavonoids, showed a significant increase, with correlation coefficients of 0.86, 0.74, and 0.88, respectively, for O_2_^−^, and 0.99, 0.94, and 0.94, respectively, for H_2_O_2_. Additionally, their VIP values all exceeded 1, indicating significant regulation of their biosynthesis by ROS, as shown in Figs 8 and 10. This regulatory effect promotes the biosynthesis of lignans.

**Fig 13:**
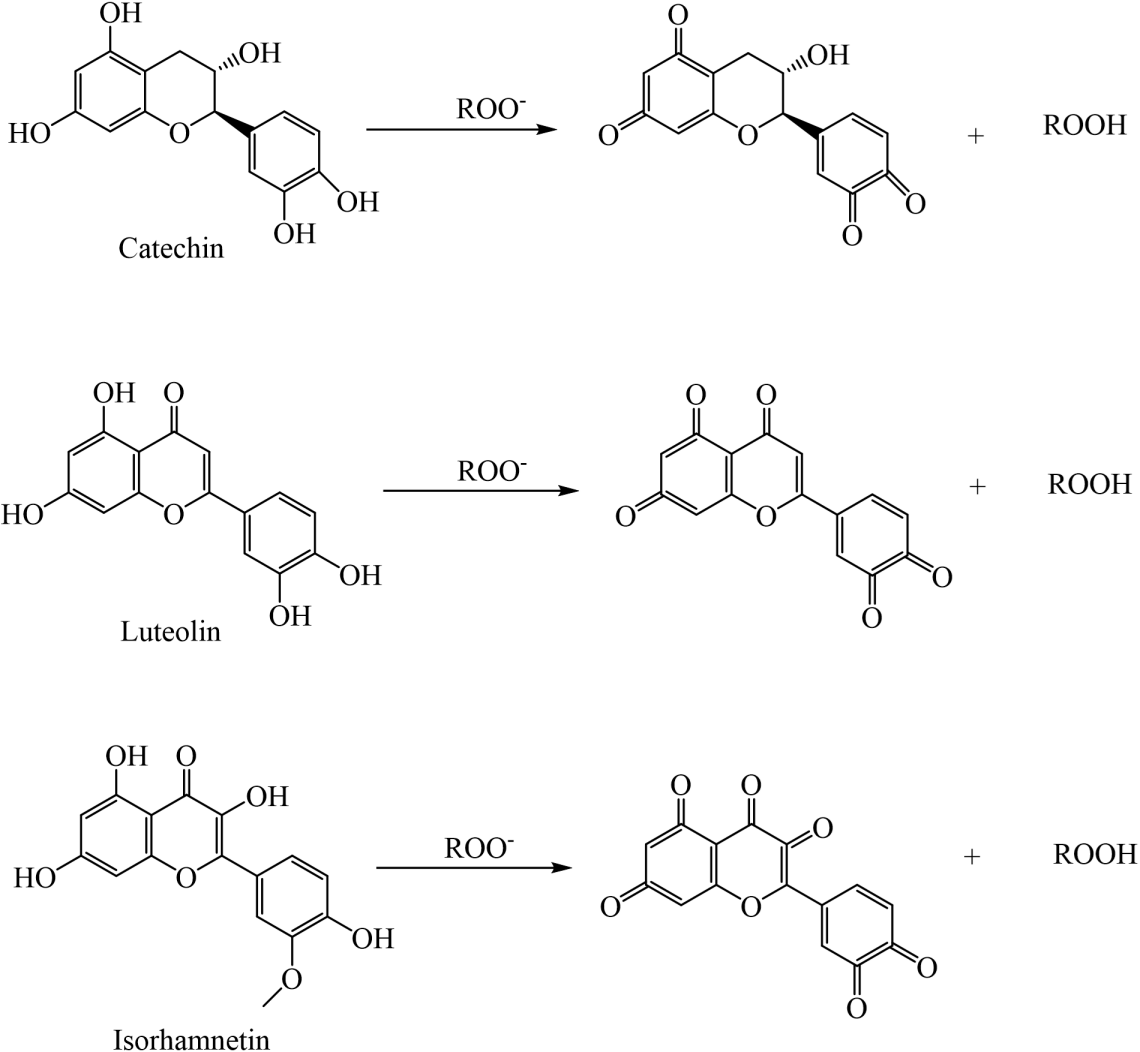
Mechanism of ROS elimination of catechins, luteolin and isorhamnetin.

**Fig 14:**
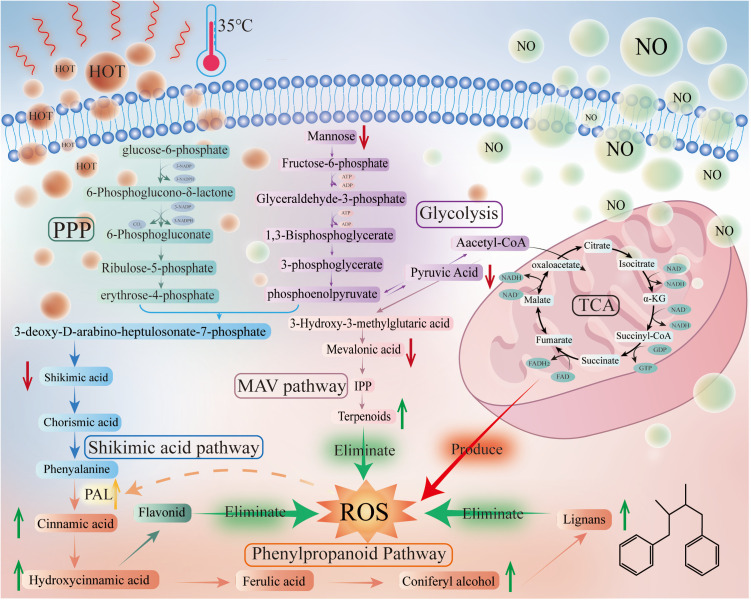
Diagram of the mechanism of metabolic changes in *Schisandrae chinensis* cells after stress. IPP is isopentenyl pyrophosphate; α-KG is α-Ketoglutaric acid (Original Fig from this study).

In the present study, a significant accumulation of flavonoids was observed. Flavonoids contain several -OH groups and have the strongest antioxidant capacity, which depends on the donation of hydrogen atoms from the -OH. Therefore, the antioxidant capacity of flavonoids is highly correlated with the position and number of -OH groups, particularly when the 3’,4’ position of B-ring is substituted with a -OH group, enhancing their biological activity [27]. Increased Catechin and Luteolin can inhibit ROS production, impede H_2_O_2_ efflux from chloroplasts, and protect DNA from oxidative damage [31], As has been shown in Fig 13.

Terpinolene consists of two isoprene units and belongs to the monoterpene group, with a strong reactive methylene group [32], capable of quenching O_2_^.^^−^, H_2_O_2_, and NO [33,34].

All these accumulated secondary metabolites have strong antioxidant capacity, enhancing cellular adaptation to environmental stresses while simultaneously improving the quality of the herbs.

### 5.3. Effects of stress on primary metabolism

The increase of secondary metabolites requires large amounts of energy and raw materials. Mannose can be converted to mannose 6-phosphate by hexokinase, which can then be isomerized to fructose 6-phosphate, subsequently entering glycolysis [35]. It has been shown that the increased ROS can lead to an increase in the activity of glycolysis-related enzymes, such as hexokinase [36]. The current study observed a reduction in mannose content, likely due to the rapid conversion of mannose to mannose 6-phosphate within the glycolytic pathway, facilitated by enhanced hexokinase activity. Glycolysis is one of the major intracellular energy-producing pathways, with many of its intermediates serving as important substrates for the synthesis of other biomolecules [35].Pyruvate is the hub linking primary and secondary metabolism. A portion of pyruvic acid can be converted to phosphoenolpyruvate, entering the shikimic acid pathway of secondary metabolism [37], and enhancing biosynthesis of Schisandrin A, Schisandrin B, Schisandrin C, Gomisin D, Gomisin G, Gomisin H, Benzoylgomisin H, Benzoylgomisin O, Angeloylgomisin P, Catechin, Isorhamnetin,Luteolin, and other lignans and flavonoids. Meanwhile, pyruvate is also converted to acetyl-CoA, which then enters the mevalonate pathway to produce terpene olefins [38]. The remaining portion of pyruvate was converted to acetyl CoA by pyruvate dehydrogenase, subsequently entering the tricarboxylic acid cycle of primary metabolism and providing energy for physiological metabolism. However, we observed no significant difference in various intermediates of the tricarboxylic acid cycle, suggesting that the primary metabolism remained largely affected by NO and high-temperature stress, hence ensuring stable energy and raw material supply for secondary metabolism.

Interestingly, the levels of pyruvate, shikimic acid, phenylalanine, and mevalonic acid significantly reduced, likely due to the enhanced activity of downstream enzymes involved in secondary metabolites synthesis, leading to their rapid conversion. PAL is an inducible enzyme located downstream of pyruvate, with its activity varying in response to ROS. Under stress conditions, the produced ROS can rapidly increase PAL activity [26], which in turn promotes the deamination of phenylalanine to form cinnamic acid [39]. The current study also showed a 69.3% increase in PAL activity (Fig 2). In addition, mevalonate kinase (MVK) is a key enzyme in the mevalonate pathway, and its activity is also regulated by ROS, showing a 2.1-fold increase under adversity [40]. In summary, the significant decreases in pyruvate, shikimic acid, phenylalanine and mevalonic acid content may result from the enhancement of secondary metabolic pathways.

The present study showed ROS have a significant increase in the early stage, followed by a rapid decrease in the subsequent stage, likely due to the action of enhanced various secondary metabolites that help maintain relatively normal and stable levels, as shown in Fig 1. The activities of PAL and MVK were closely related to ROS, with a decrease in ROS content resulting in decreased activities of both enzymes, subsequently leading to a decrease in the content of various secondary metabolites. As shown in Fig 14.

Genomics, proteomics, and metabolomics can all elucidate changes in metabolism, and the combination of multiple groups may improve knowledge of metabolic changes. However, the metabolomics technology, which focuses on alterations in secondary metabolite content, is more suitable for understanding the mechanism behind the formation of the quality of *Schisandra chinensis* herbs, potentially illuminating the mechanisms involved in herb quality development. The metabolomics shows the detection of nine ligands, including Schisandrin A, Schisandrin B, Schisandrin,Gomisin D, Gomisin G, Gomisin H, Benzoylgomisin H, Benzoylgomisin O, Angeloylgomisin P, which collectively account for 47.6%% (ΣVIP lignans/ ΣVIP total). Additionally, precursor compounds involved in lignan synthesis, including Cinnamic acid, Hydroxycinnamic acid, Coniferyl alcohol, contribute to a total of 64.9% of various components associated with lignan. Furthermore, four of the top 5 metabolites with the highest VIP scores are lignans. The key significance of lignans in Schisandrae chinensis fructus’s stress response is highlighted, since the combination of exogenous NO with high-temperature can significantly increase the lignan production via ROS, hence improving the quality of Schisandrae chinensis fructus.

In practical application, compared to traditional methods of inducing plant adversity, this approach offers significant advantages: it is simple, cost-effective, and easy to implement. Furthermore, SNP is clinically approved for the treatment of hypertension as an injectable agent, ensuring its safety profile, and the use of a low concentration (0.5 mmol/L) further enhances safety. Moreover, this method is applied exclusively to the medicinal parts post-harvest, eliminating potential impact on crop yield.

## 6. Conclusion

This work demonstrated that the combination of exogenous NO with high-temperature can induce the physiological state of Schisandrae chinensis fructus under adverse conditions. The intracellular levels of ROS are increased, leading to a redistribution of plant metabolic pathways, an enhancement of secondary metabolism, and a large number of primary metabolites for the production of secondary metabolites with higher antioxidant activity. By which the quality of the Schisandrae chinensis fructus is improved. Therefore, this study elucidates the scientific connotation of *quality generated by adversity* to a certain extent, and can lay a good theoretical foundation for improving the quality of medicinal herbs.

## Supporting information

S1 File Minimal data set. (XLSX)
